# Fibroblast Growth Factor 23 and Mortality Among Prevalent Hemodialysis Patients in the Japan Dialysis Outcomes and Practice Patterns Study

**DOI:** 10.1016/j.ekir.2020.08.013

**Published:** 2020-08-20

**Authors:** Hirotaka Komaba, Douglas S. Fuller, Masatomo Taniguchi, Suguru Yamamoto, Takanobu Nomura, Junhui Zhao, Brian A. Bieber, Bruce M. Robinson, Ronald L. Pisoni, Masafumi Fukagawa

**Affiliations:** 1 Division of Nephrology, Endocrinology, and Metabolism, Tokai University School of Medicine, Isehara, Japan; 2 The Institute of Medical Sciences, Tokai University, Isehara, Japan; 3 Arbor Research Collaborative for Health, Ann Arbor, Michigan, USA; 4 Fukuoka Renal Clinic, Fukuoka, Japan; 5 Division of Clinical Nephrology and Rheumatology, Niigata University Graduate School of Medical and Dental Sciences, Niigata, Japan; 6 Medical Affairs Department, Kyowa Kirin Co., Ltd., Tokyo, Japan

**Keywords:** FGF23, hemodialysis, kidney failure, mortality

## Abstract

**Introduction:**

Elevated fibroblast growth factor 23 (FGF23) levels have been strongly associated with mortality in the predialysis and incident hemodialysis populations, but few studies have examined this relationship in a large cohort of prevalent hemodialysis patients and in particular among persons with high dialysis vintage. To address this, we analyzed data from the Japan Dialysis Outcomes and Practice Patterns Study (J-DOPPS).

**Methods:**

We included 1122 prevalent hemodialysis patients from the J-DOPPS phase 5 (2012–2015) who had FGF23 measurements. We evaluated the association of FGF23 levels with all-cause mortality and cardiovascular composite outcome using Cox regression adjusted for potential confounders.

**Results:**

At study enrollment, median dialysis vintage was 5.8 years (interquartile range, 2.7–12.4 years) and median FGF23 level was 2113 pg/ml (interquartile range, 583–6880 pg/ml). During 3-year follow-up, 154 of the 1122 participants died. In adjusted analyses, higher FGF23 was associated with a greater hazard of death (hazard ratio per doubling of FGF23, 1.12; 95% confidence interval, 1.03–1.21); however, the association became weaker as the dialysis vintage increased and finally disappeared in the highest tertile (>9.4 years). Similar patterns of effect modification by dialysis vintage were observed for cardiovascular composite outcome and in time-dependent models.

**Conclusion:**

Elevated FGF23 was associated with mortality and cardiovascular events in prevalent hemodialysis patients, but the association was attenuated at longer dialysis vintages. This novel finding suggests that long-term hemodialysis patients may be less susceptible to the detrimental effects of FGF23 or correlated biological processes, and additional studies are needed to gain understanding of these possibilities.

## Graphical abstract

**Figure:**
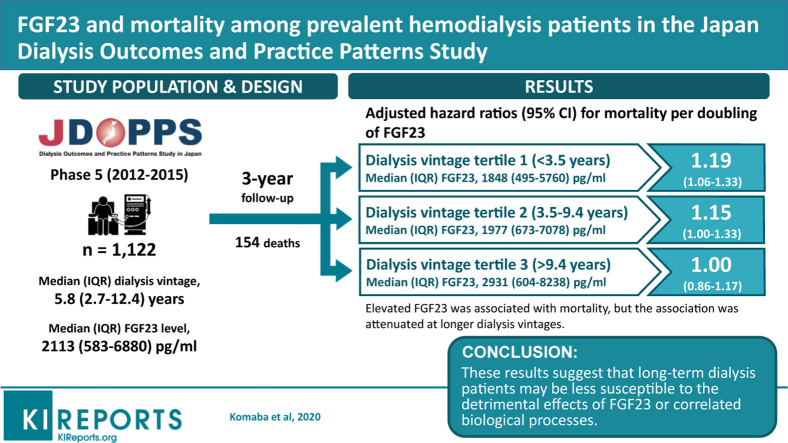

Patients with chronic kidney disease (CKD) are at high risk of mortality and cardiovascular disease, which increases as CKD progresses to kidney failure.^1^ An accumulating body of experimental and epidemiological evidence suggests that FGF23, whose circulating levels rise as kidney function declines, may contribute to the excess mortality in CKD.^2^^,^^3^ FGF23 is a bone-derived hormone that regulates phosphate and vitamin D metabolism.^4^ In predialysis CKD, elevated FGF23 helps maintain normal phosphate balance by enhancing urinary phosphate excretion, but it also causes decreased 1,25-dihydroxyvitamin D (1,25[OH]_2_D) production and thereby contributes to secondary hyperparathyroidism.^5^^,^^6^ When patients reach kidney failure, FGF23 fails to exert its physiological effects owing to loss of kidney function and systemic downregulation of Klotho,^7^^,^^8^ which form a specific receptor complex for FGF23.^9^ In this setting, extremely elevated FGF23 has been implicated in development of left ventricular hypertrophy,^10^^,^^11^ impaired leukocyte function,^12^ inflammation,^13^ and anemia,^14^ possibly in a Klotho-independent manner.

In support of these experimental data, an increasing number of observational studies have shown high FGF23 levels to be associated with death across the spectrum of CKD, including patients with non–dialysis-dependent CKD,^15^, ^16^, ^17^, ^18^, ^19^, ^20^ patients with incident hemodialysis,^21^^,^^22^ recipients of kidney transplants,^23^^,^^24^ and individuals with apparently normal kidney function.^25^, ^26^, ^27^, ^28^, ^29^, ^30^, ^31^, ^32^ However, the relationship between FGF23 and death among prevalent hemodialysis patients have been examined only in small studies^33^, ^34^, ^35^, ^36^ and few large-scale studies.^37^^,^^38^ Furthermore, these studies did not include a large number of patients with long dialysis vintage, leaving it uncertain whether the association between FGF23 and mortality exists in long-term hemodialysis patients. Therefore, the purpose of this study was to explore the association between FGF23 levels and all-cause mortality and cardiovascular events in a large cohort of prevalent hemodialysis patients, with particular focus on the differences in the association across dialysis vintage. To address this, we analyzed data from the J-DOPPS, which measured FGF23 levels as an ancillary study.

## Methods

### Data Source

The DOPPS is an international prospective cohort study of hemodialysis practices ongoing since 1996. Details on study design and methods have been published.^39^^,^^40^ We used Japanese data from DOPPS phase 5 (J-DOPPS; 2012–2015) in this analysis. Demographic and baseline clinical status variables were collected at study entry. Laboratory test values and renal medications were collected at study entry and monthly thereafter. An ancillary study to J-DOPPS 5 collected biosamples from study patients annually to ascertain laboratory values not commonly collected in dialysis practice, including FGF23. We excluded patients with missing data for demographic or renal medication covariates (*n* = 20), implausibly high FGF23 values (≥100,000 pg/ml; *n* = 6), missing 1,25(OH)_2_D or vitamin D treatment variables (*n* = 23), and missing mortality data or insufficient facility-level reporting of clinical events (*n* = 29). The final analysis sample included 1122 patients.

### Exposure and Outcomes

The primary exposure was serum FGF23, which the J-DOPPS central laboratory measured from the stored serum samples at 1-year intervals by chemiluminescence immunoassay (Hitachi Chemical Diagnostics Systems Co., Ltd., Tokyo, Japan, formerly known as Kyowa Medex Co., Ltd.), which detects the full-length, biologically intact FGF23 molecule.^41^ The central laboratory also measured calcium and phosphorus by colorimetric methods, intact parathyroid hormone (PTH) by an electrochemiluminescence immunoassay (Roche Diagnostics, Mannheim, Germany), 25-hydroxyvitamin D (25[OH]D) by chemiluminescence immunoassay (DiaSorin Inc., Stillwater, MN), 1,25(OH)_2_D by radioimmunoassay (Immunodiagnostic Systems Ltd., Boldon, UK), and high-sensitivity C-reactive protein by particle-enhanced immunonephelometry (Siemens Healthcare Diagnostic, Erlangen, Germany) annually. The first round of ancillary biosample data were collected an average of 36 days after J-DOPPS 5 study entry (range: 29–47 days) and were merged with contemporary J-DOPPS 5 data. Baseline and monthly J-DOPPS covariate data were retrieved using a date-matching window of 30 days before or after the biosample collection date. The primary outcome was all-cause mortality. We also examined a cardiovascular-related composite event, defined as the first occurrence of death or hospitalization due to cardiovascular causes (see Supplementary Table S1 for a list of qualifying factors).

### Statistical Analyses

We evaluated the association between FGF23 levels and clinical outcomes using Cox proportional hazards regressions. Time at risk for the Cox analyses began 30 days after the date of ancillary biosample collection for each patient and continued until a clinical outcome was observed (death or cardiovascular event) or departure from J-DOPPS (typically due to transfer out of the study site, 93% of non-death departures). Preliminary results suggested that a log-linear functional form for FGF23 was appropriate. Thus, we provide hazard ratios (HR) and 95% confidence intervals (CI) corresponding to a doubling of FGF23 (i.e., for a unit increase on the log_2_ scale) as well as for quartiles of FGF23 using the first quartile as the referent category. A robust variance estimator was used to account for potential intrafacility clustering. We also constructed a restricted cubic spline model^42^ to estimate a potentially nonlinear relationship between FGF23 and mortality. Sensitivity analyses included plots of risk differences for mortality by FGF23 quartiles with bootstrap 95% CIs (estimated using Austin's method^43^) and time-dependent modeling of FGF23 with mortality and cardiovascular composite outcomes.

We estimated HR and 95% CI using 3 progressive sets of potential confounders: (i) age, sex, dialysis vintage, diabetes, prior cardiovascular disease (e.g., coronary artery disease, congestive heart failure), body mass index (6% with missing values entered using missing-value indicator method), albumin, and creatinine; (ii) serum levels of albumin-corrected calcium, phosphorus, and intact PTH; (iii) prescription of active or analog vitamin D (chiefly i.v. maxacalcitol, oral alphacalcidol, and i.v./oral calcitriol in Japan). HRs for confounder variables had the expected directionality with respect to clinical outcomes. We also investigated potential interactions of FGF23 with dialysis vintage and residual kidney function (RKF; defined as 24-hour urine output >200 ml) with respect to clinical outcomes. Global tests based on Schoenfeld residuals suggested that the proportional hazards assumption was satisfied for all models. Data management and statistical analyses were performed using SAS 9.4 (SAS Institute Inc., Cary, NC).

## Results

### Description of the Study Population

At study enrollment, the median FGF23 level was 2113 pg/ml (interquartile range, 583–6880; Table 1). Among surviving patients with multiple annual measurements available during 2012–2014 (*n* = 840), the distribution of serum FGF23 levels was comparable to the full sample and remained remarkably stable over time (Supplementary Figure S1). Patients with higher FGF23 levels tended to be younger; have longer dialysis vintage, lower prevalence of diabetes, and higher levels of serum creatinine, calcium, phosphorus, and intact PTH levels; and be more often prescribed calcium-based and non–calcium-based phosphate binders and PTH-lowering medications such as active vitamin D and cinacalcet. There was no correlation between serum FGF23 with 1,25(OH)_2_D in all patients and in patients not treated with active vitamin D.

**Table 1:** Baseline characteristics, overall and by FGF23 quartile

Variable	Overall (*n* = 1122)	Quartile of FGF23			
		<574 pg/ml (*n* = 275)	574–2103 pg/ml (*n* = 285)	2104–6938 pg/ml (*n* = 285)	>6938 pg/ml (*n* = 277)
Age, y	65.5 ± 12.2	68.0 ± 10.9	66.4 ± 12.8	66.3 ± 10.9	61.3 ± 12.9
Male, %	62.2	50.9	63.5	67.4	66.8
Dialysis vintage, y	5.8 (2.7–12.4)	5.4 (2.2–12.5)	5.4 (2.6–10.1)	6.1 (2.7–12.5)	6.9 (3.2–13.5)
Residual kidney function, %	18.0	20.9	20.5	14.8	16.1
Diabetic, %	38.9	44.0	41.1	37.5	33.2
Prior cardiovascular disease, %	42.7	44.7	44.6	40.4	41.2
Body mass index, kg/m^2^	21.4 ± 3.5	20.9 ± 3.5	21.5 ± 3.8	21.5 ± 3.4	21.8 ± 3.5
Laboratory tests					
Albumin, g/dl	3.7±0.4	3.6 ± 0.4	3.7 ± 0.4	3.6 ± 0.4	3.7 ± 0.3
Creatinine, mg/dl	10.7 ± 2.8	9.1 ± 2.8	10.4 ± 2.6	11.2 ± 2.4	12.1 ± 2.7
Calcium, albumin-corrected, mg/dl	9.0 ± 0.7	8.7 ± 0.6	8.8 ± 0.6	9.0 ± 0.7	9.3 ± 0.7
Phosphorus, mg/dl	5.1 ± 1.3	4.1 ± 1.0	4.9 ± 1.0	5.3 ± 1.1	6.0 ± 1.2
Intact PTH, pg/ml	116 (60–210)	83 (44–152)	108 (60–189)	129 (64–208)	180 (84–296)
FGF23, pg/ml	2113 (583–6880)	233 (114–364)	1083 (829–1583)	3947 (2910–5295)	12411 (9322–21618)
25(OH)D, ng/ml	16.6 ± 6.3	15.0 ± 6.3	16.7 ± 5.8	17.3 ± 6.4	17.3 ± 6.6
1,25(OH)_2_D, pg/ml	13.8 ± 8.1	13.5 ± 7.9	13.8 ± 8.5	14.1 ± 7.8	13.9 ± 8.3
1,25(OH)_2_D, pg/ml^a^	10.9 ± 6.2	10.0 ± 5.5	10.3 ± 5.4	10.9 ± 6.3	13.1 ± 7.8
hsCRP, mg/dl	0.08 (0.03–0.26)	0.07 (0.03–0.27)	0.08 (0.03–0.21)	0.10 (0.04–0.29)	0.07 (0.03–0.26)
Medication, %					
Calcium-based phosphate binders	87.0	78.7	86.8	89.6	92.9
Non-calcium-based phosphate binders	59.4	45.1	54.8	60.2	75.2
Active vitamin D^b^	70.3	60.7	70.9	71.6	78.0
Cinacalcet	22.8	14.0	17.0	23.5	36.2

25(OH)D, 25-hydroxyvitamin D; 1,25(OH)_2_D, 1,25-dihydroxyvitamin D; FGF23, fibroblast growth factor 23; hsCRP, high-sensitivity C-reactive protein; PTH, parathyroid hormone.

Values indicate mean ± SD, median (interquartile range), or percentage.

a Excludes patients with active vitamin D treatment.

b Active vitamin D includes i.v. and oral forms.

### Associations of FGF23 With Clinical Outcomes

**Figure 1:**
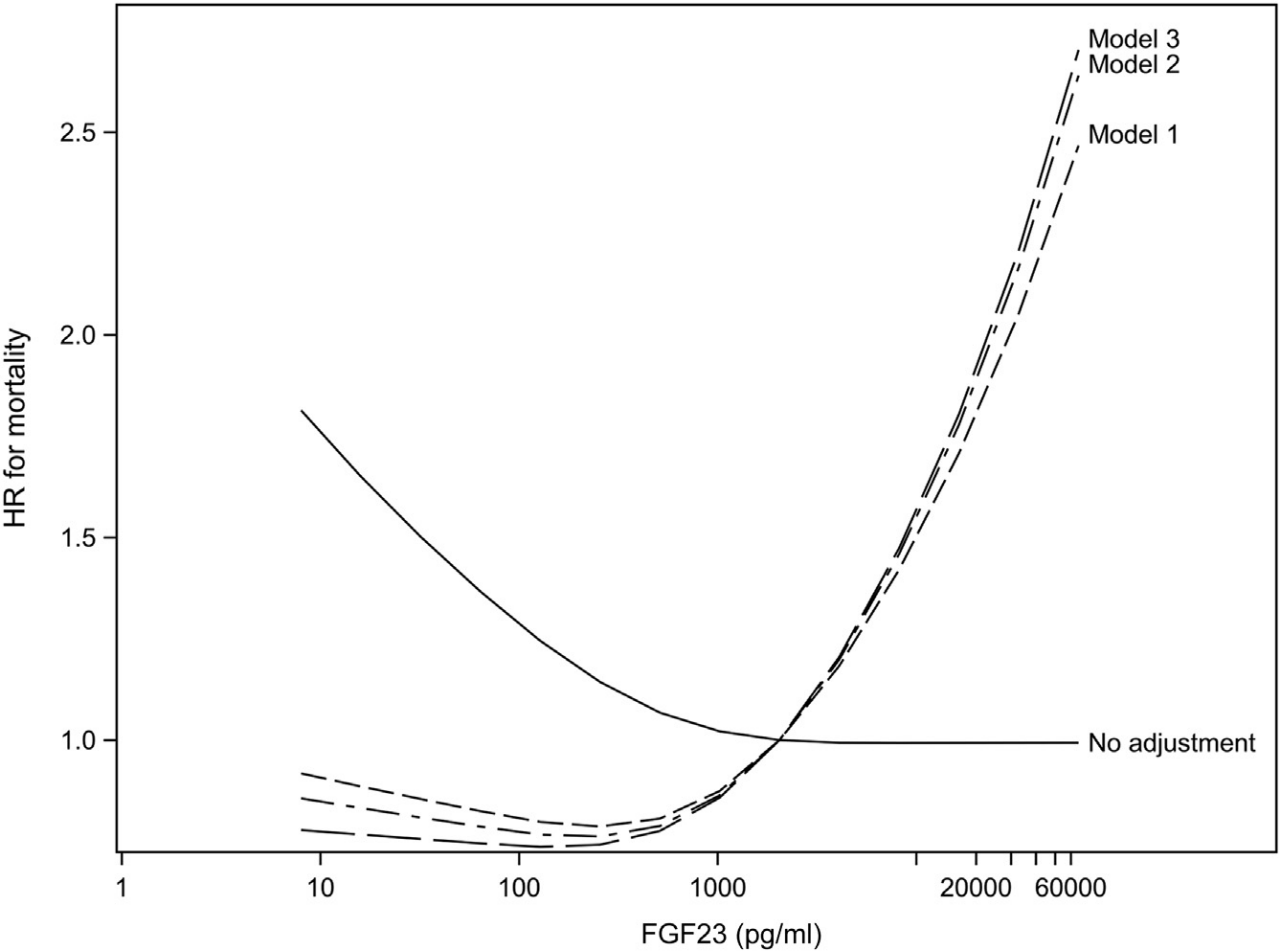
Association of fibroblast growth factor 23 (FGF23) with mortality using restricted cubic spline parameterization. Model 1 was adjusted for age, sex, dialysis vintage, diabetes, prior cardiovascular disease, body mass index, albumin, and creatinine. Model 2 was adjusted for Model 1 covariates plus calcium, phosphorus, and intact parathyroid hormone. Model 3 was adjusted for Model 2 covariates plus active vitamin D treatment. HR, hazard ratio.

Median follow-up time was 2.7 years for analyses of both mortality and cardiovascular-related events. Among the 1122 participants, 154 (13.7%; 61/1000 person-years) deaths and 167 (14.9%; 70/1000 person-years) cardiovascular-related events were observed. In the unadjusted model, no association was observed between FGF23 levels and hazard of all-cause mortality (Table 2). After adjustment for Model 1 covariates, higher FGF23 was associated with an increased hazard of mortality (HR per doubling of FGF23: 1.12; 95% CI: 1.03–1.21), although the association was not strictly linear (Figure 1). Compared with patients in the first FGF23 quartile, the risk differences for mortality at 1 year were 3.8% (95% CI: 1.4–6.6) for quartile 2, 3.1% (95% CI: 0.3–5.9) for quartile 3, and 5.8% (95% CI: 2.7–9.3) for quartile 4 (Supplementary Figure S2). Additional adjustment for markers of mineral metabolism (Model 2) and active vitamin D treatment (Model 3) yielded qualitatively similar associations. Qualitatively similar estimates patterns were obtained when allowing FGF23 and covariate values to be updated during follow-up (Supplementary Table S2).

**Table 2:** Adjusted HR (95% CI) for mortality and cardiovascular composite event, by log FGF23 and quartile of FGF23

Model	Per doubling of FGF23	Quartile of FGF23			
		<574 pg/ml	574–2103 pg/ml	2104–6938 pg/ml	>6938 pg/ml
HR (95% CI) for mortality					
No adjustment	0.97 (0.91–1.04)	1 (ref)	1.25 (0.79–1.98)	1.02 (0.68–1.52)	0.88 (0.54–1.41)
Model 1	1.12 (1.03–1.21)	1 (ref)	1.97 (1.26–3.08)	1.77 (1.10–2.83)	2.45 (1.43–4.20)
Model 2	1.12 (1.01–1.24)	1 (ref)	1.99 (1.27–3.12)	1.80 (1.07–3.03)	2.53 (1.47–4.34)
Model 3	1.13 (1.02–1.25)	1 (ref)	2.03 (1.30–3.17)	1.86 (1.10–3.12)	2.61 (1.53–4.46)
HR (95% CI) for cardiovascular composite event					
No adjustment	1.02 (0.95–1.10)	1 (ref)	1.33 (0.87–2.05)	1.26 (0.77–2.06)	1.18 (0.67–2.06)
Model 1	1.07 (0.98–1.16)	1 (ref)	1.40 (0.93–2.11)	1.49 (0.91–2.44)	1.57 (0.86–2.85)
Model 2	1.05 (0.94–1.16)	1 (ref)	1.33 (0.86–2.05)	1.37 (0.77–2.43)	1.38 (0.69–2.78)
Model 3	1.06 (0.95–1.18)	1 (ref)	1.38 (0.89–2.14)	1.44 (0.79–2.64)	1.48 (0.72–3.05)

CI, confidence interval; FGF23, fibroblast growth factor 23; HR, hazard ratio.

Model 1 adjusted for age, sex, dialysis vintage, diabetes, prior cardiovascular disease, body mass index, albumin, and creatinine.

Model 2 adjusted for Model 1 covariates plus calcium, phosphorus, and intact parathyroid hormone.

Model 3 adjusted for Model 2 covariates plus active vitamin D treatment.

There was also a trend toward an association between elevated FGF23 and cardiovascular-related events in the base model (Model 1) (HR per doubling of FGF23: 1.07; 95% CI: 0.98–1.16), although the association was not statistically significant. This relationship was qualitatively unchanged after adjustment for markers of mineral metabolism (Model 2) and active vitamin D treatment (Model 3).

### Interactions With Dialysis Vintage and RKF

Patients with shorter dialysis vintage tended to have lower FGF23 levels (lower median and smaller interquartile range), but the association between FGF23 and mortality in the base model (Model 1) was strongest among patients with <3.5 years on dialysis (HR per doubling of FGF23: 1.19; 95% CI: 1.06–1.33; Table 3), modestly weaker among patients with 3.5 to 9.4 years on dialysis (HR per doubling of FGF23: 1.15; 95% CI: 1.00–1.33), and essentially absent among patients with >9.4 years on dialysis (HR per doubling of FGF23: 1.00; 95% CI: 0.86–1.17). These associations were unaltered by further adjustment for covariates in Models 2 and 3 or additional adjustment for RKF (Supplementary Table S3). Qualitatively similar interactions were observed for the cardiovascular-related composite outcome and in time-dependent models (Supplementary Table S4). Similarly, patients with RKF tended to have lower FGF23 levels, but their mortality association (HR per doubling of FGF23: 1.32; 95% CI: 1.12–1.55) was much stronger compared with patients without RKF (HR per doubling of FGF23: 1.09; 95% CI: 0.99–1.19; Table 4).

**Table 3:** Adjusted HR (95% CI) for mortality and cardiovascular composite event, interaction of log FGF23 with tertile of dialysis vintage

Model	Tertile of dialysis vintage			*P* for interaction
	<3.5 y (*n* = 377)	3.5–9.4 y (*n* = 376)	>9.4 y (*n* = 369)	
Median FGF23 (IQR), pg/ml	1848 (495–5760)	1977 (673–7078)	2931 (604–8238)	-
HR (95% CI) for mortality per doubling of FGF23				
No adjustment	1.00 (0.90–1.12)	1.01 (0.90–1.13)	0.91 (0.80–1.04)	0.38
Model 1	1.19 (1.06–1.33)	1.15 (1.00–1.33)	1.00 (0.86–1.17)	0.16
Model 2	1.19 (1.04–1.36)	1.15 (0.97–1.36)	1.00 (0.85–1.17)	0.15
Model 3	1.20 (1.05–1.37)	1.16 (0.98–1.37)	1.01 (0.86–1.18)	0.15
HR (95% CI) for cardiovascular composite event per doubling of FGF23				
No adjustment	1.08 (0.97–1.20)	1.09 (0.97–1.21)	0.94 (0.86–1.03)	0.05
Model 1	1.15 (1.04–1.28)	1.16 (1.03–1.31)	0.96 (0.87–1.06)	0.008
Model 2	1.13 (1.00–1.27)	1.13 (0.99–1.30)	0.94 (0.83–1.06)	0.008
Model 3	1.14 (1.01–1.29)	1.15 (0.99–1.32)	0.95 (0.84–1.08)	0.008

CI, confidence interval; FGF23, fibroblast growth factor 23; HR, hazard ratio; IQR, interquartile range.

Model 1 adjusted for age, sex, diabetes, prior cardiovascular disease, body mass index, albumin, and creatinine.

Model 2 adjusted for Model 1 covariates plus calcium, phosphorus, and intact parathyroid hormone.

Model 3 adjusted for Model 2 covariates plus active vitamin D treatment.

**Table 4:** Adjusted HR (95% CI) for mortality and cardiovascular composite event, interaction of log FGF23 with residual kidney function

Model	Residual kidney function		*P* for interaction
	Urine output >200 ml/d (*n* = 172)	Urine output ≤200 ml/d (*n* = 781)	
Median FGF23 (IQR), pg/ml	1629 (509–5891)	2233 (604–6902)	-
HR (95% CI) for mortality per doubling of FGF23			
No adjustment	1.06 (0.89–1.26)	0.95 (0.90–1.02)	0.29
Model 1	1.32 (1.12–1.55)	1.09 (0.99–1.19)	0.03
Model 2	1.31 (1.09–1.58)	1.08 (0.97–1.20)	0.02
Model 3	1.33 (1.10–1.60)	1.09 (0.98–1.20)	0.02
HR (95% CI) for cardiovascular composite event per doubling of FGF23			
No adjustment	1.09 (0.91–1.31)	1.01 (0.94–1.08)	0.40
Model 1	1.15 (0.94–1.41)	1.05 (0.97–1.14)	0.38
Model 2	1.13 (0.92–1.39)	1.03 (0.92–1.14)	0.35
Model 3	1.15 (0.94–1.41)	1.04 (0.93–1.16)	0.33

CI, confidence interval; FGF23, fibroblast growth factor 23; HR, hazard ratio; IQR, interquartile range.

Model 1 adjusted for age, sex, dialysis vintage, diabetes, prior cardiovascular disease, body mass index, albumin, and creatinine.

Model 2 adjusted for Model 1 covariates plus calcium, phosphorus, and intact parathyroid hormone.

Model 3 adjusted for Model 2 covariates plus active vitamin D treatment.

## Discussion

In this prospective cohort of 1122 Japanese prevalent hemodialysis patients in the DOPPS, we confirmed that elevated FGF23 levels were associated with increased hazard of death. However, the association between FGF23 and mortality became weaker as the duration of dialysis increased and finally disappeared in the highest dialysis vintage tertile (>9.4 years), even though average FGF23 levels were highest in this vintage tertile. Similar patterns of effect modification by dialysis vintage were observed for cardiovascular composite outcome. Likewise, the associations between FGF23 and outcomes were less pronounced in patients without RKF, who had higher FGF23 levels than patients with RKF. To our knowledge, this is the first to characterize the interactions among FGF23, clinical outcome, and dialysis vintage; we could address this question by using data from the J-DOPPS, which included a sufficient number of patients with high dialysis vintage. These novel findings provide a new perspective on the relationship between FGF23 and mortality and generate new questions and hypotheses on the possible toxic effects of FGF23.

Prior epidemiological studies have consistently shown a strong association between elevated FGF23 and mortality in non–dialysis-dependent patients with CKD,^15^, ^16^, ^17^, ^18^, ^19^, ^20^ incident hemodialysis patients,^21^^,^^22^ kidney transplant recipients,^23^^,^^24^ and individuals with apparently normal kidney function.^25^, ^26^, ^27^, ^28^, ^29^, ^30^, ^31^, ^32^ By contrast, limited data exist on the association between FGF23 and mortality in a large cohort of prevalent hemodialysis patients. In secondary analyses of the HEMO study^37^ and the EVOLVE (Evaluation of Cinacalcet Hydrochloride Therapy to Lower Cardiovascular Events) trial,^38^ elevated FGF23 levels were associated with greater risk of cardiovascular events and mortality in adjusted analysis. However, the magnitude of these associations was not as strong as the studies of predialysis and incident hemodialysis patients. Furthermore, such associations were not observed in unadjusted analysis in the HEMO study, which contrasts with the studies of predialysis and incident hemodialysis patients.

Like the results of these 2 large-scale studies, our study found associations of elevated FGF23 with mortality and cardiovascular events, but these relationships were modest and became significant only after multivariate adjustment. Several smaller studies have also examined this relationship in maintenance hemodialysis patients, but the results have been inconsistent.^33^, ^34^, ^35^, ^36^ Of note, a recent ancillary study of the Chronic Renal Insufficiency Cohort (CRIC) study^44^ demonstrated that individuals with rapidly rising FGF23 trajectories were at >15-fold higher risk of death than those with stable FGF23 levels. A similar pattern of association was reported from the HEMO study,^45^ but the effect size was far smaller than that of the CRIC study. In aggregate, it appears that an association between elevated FGF23 and mortality could exist in prevalent hemodialysis patients, but to a lesser extent compared with the predialysis and incident hemodialysis populations.

In this context, it is interesting to note that in the present study of prevalent hemodialysis patients, the associations of elevated FGF23 with mortality and cardiovascular events became weaker as the duration of dialysis increased and finally disappeared among patients on long-term dialysis. This finding provides a clue to help understand the inconsistency in the strength of the association of FGF23 with mortality between incident and prevalent hemodialysis patients. Together with the findings from previous studies,^15^, ^16^, ^17^, ^18^, ^19^, ^20^, ^21^, ^22^, ^23^, ^24^ it could be concluded that the association of FGF23 with mortality persists to a large degree during the predialysis period, starts to be progressively attenuated after the initiation of dialysis, and almost disappears during long-term dialysis, but resurges after kidney transplantation.

As most maintenance hemodialysis patients have much higher FGF23 levels than non–dialysis-dependent patients with CKD, it might be surprising that the magnitude of the association between elevated FGF23 and mortality is not greater in prevalent dialysis patients than nondialyzed patients with CKD. These findings are in keeping with the results of a recent meta-analysis^46^ and may not support a direct cause-effect relationship between FGF23 and mortality. However, it is also possible that another mechanism modifies the association of FGF23 with mortality in hemodialysis patients.

One possibility is that kidney function mediates the association between elevated FGF23 and mortality. Although it remains an open question whether FGF23 directly contributes to kidney injury, elevated FGF23 levels have been associated with progression of CKD^15^^,^^16^^,^^47^ and acute kidney injury.^48^^,^^49^ As kidney function strongly influences the risk of mortality and cardiovascular disease,^1^ the greater hazard of mortality in association with elevated FGF23 in nondialyzed CKD patients might in part be explained by decline in kidney function. Likewise, in patients undergoing hemodialysis, preservation of RKF is associated with better clinical outcomes^50^; thus, elevated FGF23 might be associated with mortality and cardiovascular events through decline in RKF. If so, the presence of RKF could be an effect modifier of the association of FGF23 with mortality and cardiovascular disease, which may explain the difference in the hazard associated with elevated FGF23 between incident versus prevalent hemodialysis patients, and between short-term versus long-term hemodialysis patients, as well as between hemodialysis patients with versus without RKF. However, it should be noted that the effect modification by dialysis vintage persisted even after adjustment for RKF, suggesting that RKF alone cannot fully explain the interaction among FGF23, mortality, and dialysis vintage.

Another possibility is a selection effect introduced by recruiting prevalent hemodialysis patients, who have survived over many years of CKD and after initiation of dialysis, during which progressively rising FGF23 levels are strongly associated with mortality.^15^, ^16^, ^17^, ^18^, ^19^, ^20^, ^21^^,^^44^ This argument asserts that prevalent hemodialysis patients, particularly those with longer dialysis vintage, are a selected population who are less susceptible to the detrimental effects of FGF23 or correlated biological processes, either intrinsically or extrinsically. However, this possibility does not fit the observation that elevated FGF23 was associated with mortality in recipients of kidney transplants,^23^^,^^24^ most of whom were survivors over a long period of CKD and kidney failure.

In this regard, it is intriguing to speculate that the toxic effects of FGF23 are operating in patients with kidney failure but become attenuated during long-term uremia. One may speculate that the systemic Klotho deficiency in kidney failure should promote the binding of FGF23 to FGF receptors of the nonclassical target organs that do not express Klotho and thereby exacerbate the toxicity of FGF23.^17^ However, this hypothesis remains to be determined, and there might be another mechanism that attenuates the toxic effects of FGF23. For instance, some uremic toxins might interfere with the effects of FGF23, in a manner analogous to erythropoietin resistance in renal anemia and skeletal resistance to PTH in low-turnover bone disease. Of note, this possibility is in keeping with the observation that the association between elevated FGF23 and the hazard of mortality resurges after kidney transplant.^22^^,^^23^ Additional studies are required to explore these possibilities.

Given the body of observational and supportive experimental evidence linking FGF23 and adverse outcomes, and the recent development of neutralizing anti-FGF23 antibodies^51^ and blocking antibodies against FGF receptor 4 that mediates the pathologic effects of FGF23,^11^^,^^13^ there is a growing interest to test whether interventions targeting FGF23 improve clinical outcomes in patients with kidney failure. In this context, our findings that the hazard of death and cardiovascular events associated with elevated FGF23 was attenuated at longer dialysis vintages may have important implications for the design of future clinical trials targeting FGF23. If our findings are confirmed in other cohorts and experimental studies provide plausible explanation for the attenuation of FGF23 toxicity or its correlated process during long-term uremia, future clinical trials of FGF23-targeted therapy should focus on incident hemodialysis patients rather than prevalent hemodialysis patients. Importantly, mortality rates in the first few months following initiation of hemodialysis are substantially higher,^52^ which further strengthens the justification of recruiting incident patients in these clinical trials.

A unique strength of the present study is that the study population was restricted to Japanese hemodialysis patients, who have lower mortality rate and less chance of kidney transplantation compared with individuals in other regions. This allowed us to include a large number of long-term hemodialysis patients and examine the interaction with dialysis vintage. Other strengths of the study include the comprehensive data collection in the DOPPS and the central measurement of key biochemical markers including FGF23 that was done as an ancillary study of the J-DOPPS. There are also several important limitations. First, as in other cohort studies, we were unable to determine whether the association between elevated FGF23 and mortality is causal and could be explained by the toxic effects of FGF23 as implicated by experimental studies.^10^, ^11^, ^12^, ^13^, ^14^ Second, the attenuation of the association of FGF23 with mortality and cardiovascular events in patients with longer dialysis vintage might be explained by a selection effect, as discussed previously, and should be interpreted with caution. Understanding the mechanism underlying this observation must await carefully designed future studies. Third, we did not measure C-terminal FGF-23 levels, which might provide additional information on the regulation of FGF23 production. However, it is reported that virtually all circulating FGF23 is full-length in dialysis patients.^53^ Finally, the restriction of the study population to Japanese hemodialysis patients can be regarded as a strength of this study, as noted previously, but may limit generalizability to other populations. For example, prior studies found greater risk of death associated with elevated FGF23 in black compared with white individuals.^21^^,^^22^ Additional studies in the populations worldwide are needed to confirm our findings.

In conclusion, using the J-DOPPS cohort, we demonstrated that elevated FGF23 levels were associated with increased hazard of death and cardiovascular events in prevalent hemodialysis patients. However, the hazard of death and cardiovascular events associated with elevated FGF23 was attenuated at longer dialysis vintages and finally disappeared among persons on dialysis for more than 10 years. Similarly, the association was less pronounced in patients without RKF compared with patients with RKF. These novel findings suggest that patients undergoing long-term hemodialysis or those with anuria are less susceptible to the detrimental effects of FGF23 or correlated biological processes, and additional studies are needed to evaluate these possibilities.

## Disclosure

HK has received honoraria, consulting fees, and/or grant support from Bayer Yakuhin, Chugai Pharmaceutical, Japan Tobacco, Kyowa Kirin, Novartis, and Ono Pharmaceutical. MT has received consulting fees from Kyowa Kirin. TN is an employee of Kyowa Kirin. SY has received honoraria from Kyowa Kirin. MF has received honoraria, consulting fees, and/or grant support from Bayer Yakuhin, Fresenius Kabi, Kissei Pharmaceutical, Kyowa Kirin, Ono Pharmaceutical, and Torii Pharmaceutical. DSF, JZ, BAB, BMR, and RLP are employees for the nonprofit research organization Arbor Research Collaborative for Health, which has designed and carries out the DOPPS Program. Grants are made to Arbor Research Collaborative for Health and not to individual investigators.
