# Supplementary material for: Fibroblast Growth Factor 23 and Mortality Among Prevalent Hemodialysis Patients in the Japan Dialysis Outcomes and Practice Patterns Study
Source: Kidney Int Rep. 2020 Aug 20;5(11):1956–64. doi: 10.1016/j.ekir.2020.08.013 (PMC7609896; doi:10.1016/j.ekir.2020.08.013)
Supplement: Supplementary File (PDF) [file mmc1.pdf]

Table S1. Qualifying conditions for composite cardiovascular event.

Causes of death	Hospital diagnoses	Hospital procedures
• Atherosclerotic heart disease<br>• Cardiac arrest, cause unknown<br>• Cardiac arrhythmia<br>• Cardiomyopathy<br>• Congestive heart failure<br>• Mesenteric infarction/ischemic bowel<br>• Myocardial infarction, acute<br>• Pericarditis, incl. cardiac tamponade<br>• Pulmonary edema due to exogenous fluid<br>• Stroke, hemorrhagic<br>• Stroke, ischemic	• Acute myocardial infarction (MI)<br>• Angina<br>• Cardiac arrest/sudden death<br>• Cardiomyopathy<br>• Congestive heart failure	• Cardiac catheterization<br>• Coronary angioplasty<br>• Coronary artery bypass graft

Table S2. Adjusted HR (95% CI) for mortality, by log FGF23 and quartile of FGF23 (time-dependent model).

	Per doubling of FGF23	Quartile of FGF23			
		<574 pg/ml	574-2103 pg/ml	2104-6938 pg/ml	>6938 pg/ml
No adjustment	0.97 (0.90-1.03)	1 (ref)	1.10 (0.67-1.80)	0.89 (0.60-1.33)	0.90 (0.57-1.44)
Model 1	1.13 (1.04-1.22)	1 (ref)	1.47 (0.87-2.48)	1.57 (0.97-2.54)	2.55 (1.49-4.36)
Model 2	1.08 (0.99-1.18)	1 (ref)	1.37 (0.82-2.32)	1.39 (0.82-2.36)	2.12 (1.27-3.55)
Model 3	1.09 (1.00-1.19)	1 (ref)	1.40 (0.83-2.36)	1.43 (0.83-2.47)	2.19 (1.28-3.76)

CI, confidence interval; FGF23, fibroblast growth factor 23; HR, hazard ratio.

Model 1 adjusted for age, sex, dialysis vintage, diabetes, prior cardiovascular disease, body mass index, albumin, and creatinine.

Model 2 adjusted for Model 1 covariates plus calcium, phosphorus, and intact PTH.

Model 3 adjusted for Model 2 covariates plus active vitamin D treatment.

Table S3. Adjusted HR (95% CI) for mortality and cardiovascular composite event, interaction of log FGF23 with tertile of dialysis vintage (RKF-adjusted model).

	Tertile of dialysis vintage			
	<3.5 years (n=377)	3.5-9.4 years (n=376)	>9.4 years (n=369)	*P* for interaction
Median FGF23 (IQR), pg/ml	1848 (495-5760)	1977 (673-7078)	2931 (604-8238)	-
HR (95% CI) for mortality per doubling of FGF23				
No adjustment	1.00 (0.90-1.12)	1.01 (0.90-1.13)	0.91 (0.80-1.04)	0.38
Model 1 + RKF	1.18 (1.05-1.33)	1.15 (1.00-1.33)	1.00 (0.86-1.16)	0.16
Model 2 + RKF	1.18 (1.04-1.36)	1.15 (0.96-1.37)	0.99 (0.84-1.16)	0.15
Model 3 + RKF	1.19 (1.04-1.37)	1.16 (0.97-1.37)	1.00 (0.85-1.17)	0.15
HR (95% CI) for cardiovascular composite event per doubling of FGF23				
No adjustment	1.08 (0.97-1.20)	1.09 (0.97-1.21)	0.94 (0.86-1.03)	0.05
Model 1 + RKF	1.15 (1.03-1.29)	1.16 (1.03-1.31)	0.96 (0.87-1.06)	0.01
Model 2 + RKF	1.13 (1.00-1.27)	1.13 (0.98-1.31)	0.94 (0.83-1.06)	0.009
Model 3 + RKF	1.15 (1.02-1.29)	1.15 (0.99-1.33)	0.95 (0.84-1.08)	0.008

CI, confidence interval; FGF23, fibroblast growth factor 23; HR, hazard ratio; IQR, interquartile range; RKF, residual kidney function.

Model 1 + RKF adjusted for age, sex, diabetes, prior cardiovascular disease, body mass index, albumin, creatinine, and RKF (urine output >200 ml/day, ≤200 ml/day, and missing).

Model 2 + RKF adjusted for Model 1 + RKF covariates plus calcium, phosphorus, and intact PTH.

Model 3 + RKF adjusted for Model 2 + RKF covariates plus active vitamin D treatment.

Table S4. Adjusted HR (95% CI) for mortality, interaction of log FGF23 with tertile of dialysis vintage (time-dependent model).

	Tertile of dialysis vintage			
	<3.5 years (n=377)	3.5-9.4 years (n=376)	>9.4 years (n=369)	*P* for interaction
Median FGF23 (IQR), pg/mL	1848 (495-5760)	1977 (673-7078)	2931 (604-8238)	-
HR (95% CI) for mortality per doubling of FGF23				
No adjustment	1.03 (0.93-1.14)	0.95 (0.84-1.07)	0.92 (0.80-1.05)	0.39
Model 1	1.25 (1.12-1.40)	1.08 (0.94-1.24)	1.02 (0.87-1.20)	0.05
Model 2	1.20 (1.07-1.36)	1.03 (0.89-1.19)	0.97 (0.84-1.14)	0.04
Model 3	1.21 (1.07-1.37)	1.04 (0.89-1.20)	0.98 (0.84-1.14)	0.04

Model 1 adjusted for age, sex, diabetes, prior cardiovascular disease, body mass index, albumin, and creatinine.

Model 2 adjusted for Model 1 covariates plus calcium, phosphorus, and intact PTH.

Model 3 adjusted for Model 2 covariates plus active vitamin D treatment.

Figure S1. Distributions of FGF23 by collection year, among patients with FGF23 measured each year (n=840).

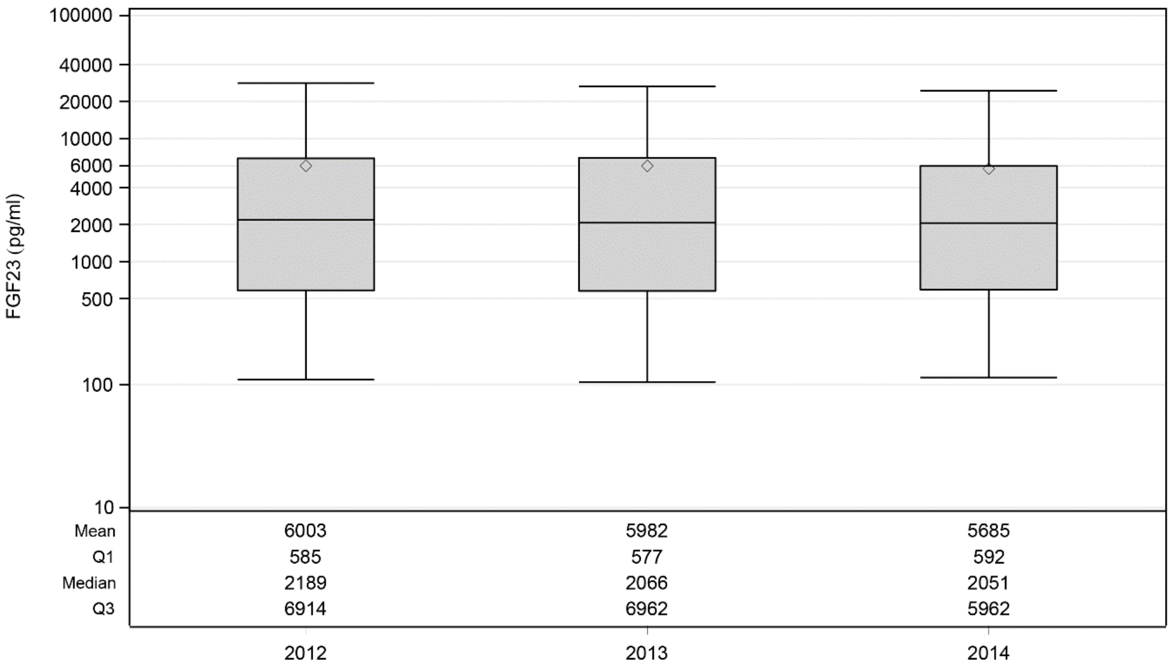

The diamond shape denotes the mean; whiskers extend to 5th and 95th percentiles.

Figure S2. Risk differences for mortality between FGF23 quartiles.

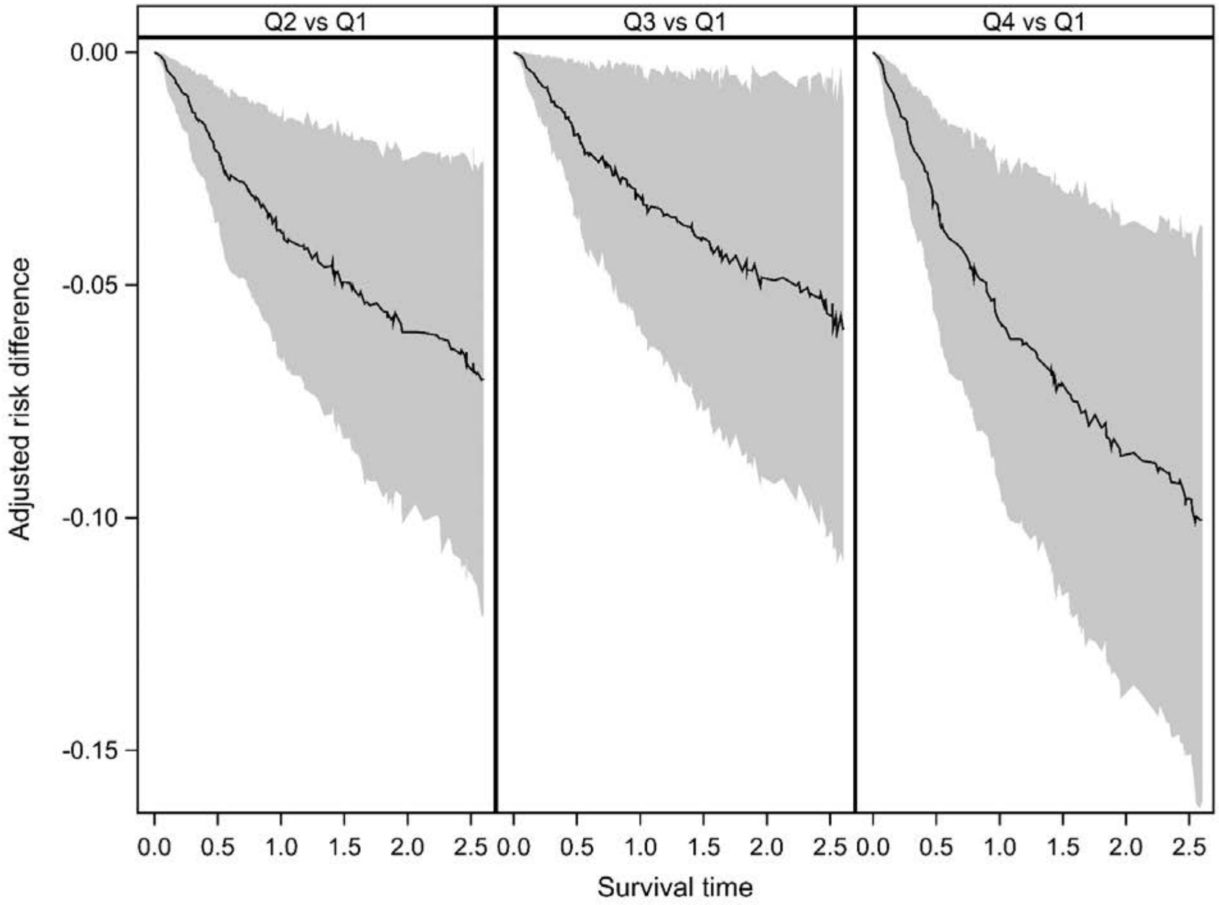

Mean risk differences (direct-adjusted for Model 1 covariates) and 95% confidence intervals are estimated from 1000 bootstrap replicates.
